# Early warning signal for dengue outbreaks and identification of high risk areas for dengue fever in Colombia using climate and non-climate datasets

**DOI:** 10.1186/s12879-017-2577-4

**Published:** 2017-07-10

**Authors:** Jung-Seok Lee, Mabel Carabali, Jacqueline K. Lim, Victor M. Herrera, Il-Yeon Park, Luis Villar, Andrew Farlow

**Affiliations:** 1 Department of Zoology, The University of Oxford, The Tinbergen Building, South Parks Road, Oxford, OX1 3PS UK; 2 Department of Epidemiology, McGill University, Biostatistics and Occupational Health, Purvis Hall, 1020 Pine Avenue West, Quebec, Montreal H3A1A2 Canada; 3 International Vaccine Institute, SNU Research Park, San 4-8, Seoul, Nakseongdae-dong, Gwanak-gu 151-919 South Korea; 4 Clinical Epidemiology Unit, School of Medicine, Universidad Industrial de Santander, Cra 32 # 29 - 31 Office, 304 Bucaramanga, Santander Colombia

**Keywords:** Dengue, Early warning system, Dengue epidemic, Population at risk for dengue fever

## Abstract

**Background:**

Dengue has been prevalent in Colombia with high risk of outbreaks in various locations. While the prediction of dengue epidemics will bring significant benefits to the society, accurate forecasts have been a challenge. Given competing health demands in Colombia, it is critical to consider the effective use of the limited healthcare resources by identifying high risk areas for dengue fever.

**Methods:**

The Climate Risk Factor (CRF) index was constructed based upon temperature, precipitation, and humidity. Considering the conditions necessary for vector survival and transmission behavior, elevation and population density were taken into account. An Early Warning Signal (EWS) model was developed by estimating the elasticity of the climate risk factor function to detect dengue epidemics. The climate risk factor index was further estimated at the smaller geographical unit (5 km by 5 km resolution) to identify populations at high risk.

**Results:**

From January 2007 to December 2015, the Early Warning Signal model successfully detected 75% of the total number of outbreaks 1 ~ 5 months ahead of time, 12.5% in the same month, and missed 12.5% of all outbreaks. The climate risk factors showed that populations at high risk are concentrated in the Western part of Colombia where more suitable climate conditions for vector mosquitoes and the high population level were observed compared to the East.

**Conclusions:**

This study concludes that it is possible to detect dengue outbreaks ahead of time and identify populations at high risk for various disease prevention activities based upon observed climate and non-climate information. The study outcomes can be used to minimize potential societal losses by prioritizing limited healthcare services and resources, as well as by conducting vector control activities prior to experiencing epidemics.

**Electronic supplementary material:**

The online version of this article (doi:10.1186/s12879-017-2577-4) contains supplementary material, which is available to authorized users.

## Background

Dengue is complicated. There are four serotypes of the dengue virus, and dengue infection occurs in almost all age groups [1, 2]. Dengue is endemic in many parts of the tropics and subtropics, and dengue endemic countries are also exposed to the risk of periodic outbreaks [1, 3]. In Colombia, dengue has been prevalent over the last 20 years with different degrees of incidence rates and epidemics in various geographical locations [4, 5]. Due to the complexity of the disease, there are still large knowledge gaps regardingthe causes of dengue epidemics [6–9]. Infection with one serotype provides life-long immunity to that specific serotype. Therefore, subsequent introduction of the same serotype in a community would be less likely to cause the occurrence of a dengue epidemic if there were a small population of dengue-susceptible individuals [7, 8, 10]. However, due to a high degree of antigenic cross-reactivity, sequential infection of two different serotypes can bring favorable or detrimental outcomes depending upon known and unknown factors including timing of infection [2, 11, 12]. For example, a primary infection may help slow the spread of secondary heterologous infection when some degrees of cross-protection are conferred [2, 12–14]. On the other hand, many studies have shown that subsequent heterologous infection would likely increase the probability of experiencing severe dengue fever [15–18]. One of the known mechanisms is the antibody dependent enhancement (ADE) during the second infection mediated by non-protective heterotypic antibodies arising from the primary infection [2, 11, 14, 19]. In dengue endemic countries such as Colombia, the number of dengue cases is periodically reported to the upper- level health management unit (i.e. provincial or Ministry of Health) from various health facilities at the municipality level [4, 20]. In the case of dengue fever, like any other diseases, severe cases are detected more easily than mild symptoms, which in turn, leads to a higher volume of reported caseload [21]. Thus, having more severe cases is also related to the high likelihood of observing dengue epidemics when an epidemic is determined based on official statistics of reported cases.

While it is undeniable that all of these aspects would affect the occurrence of dengue epidemics directly and indirectly, it does not appear to be practical in proving the impacts of these factors on the occurrence of dengue epidemics due to the following reasons: (1) despite various efforts to disentangle the complexity of the disease [11], it is still uncertain to generalize how one serotype reacts with another in terms of cross-protection or ADE for all possible scenarios among four serotypes, as well as the duration of the interactions [22, 23]; (2) even if this uncertainty is going to be uncovered in the near future, it would be very difficult to obtain the details of sero-prevalence history over a long period of time for each cohort in all specific locations. These limitations make it difficult to understand how much of each factor would contribute to the actual probability of a dengue epidemic occurrence [7, 9, 24].

A more practical way is to focus on the basic principle of the occurrence of a dengue epidemic. Simply put, a dengue epidemic occurs when a large number of people become infected within a short period of time [2, 7]. It requires a large number of vector mosquitoes (*Aedes aegypti*), as well as high transmission probability, and frequent contact between people and the vectors (biting rate) to sustain transmission [2, 3, 7]. In other words, a dengue epidemic would more likely occur when vector mosquitoes increase within a short time period in a location where dengue viruses are currently circulating and population density with no immunity to one of the four serotypes is high during the same period [8, 9, 24]. Further, the importation of infected cases into a community where there is no immunity to that specific serotype would cause an epidemic as well.

Following this principle, the main concept of this study lies in the increase of vector mosquitoes as a primary factor of a dengue epidemic taking into account population density at different elevation levels. As a vector-borne viral disease, there is a wide range of factors that influence the spatial and temporal dynamics of mosquito populations: temperature, rainfall, and humidity, etc. [9, 24, 25]. There have been several efforts to understand the relationship between dengue epidemics and climate change. Juffrie and Focks used sea surface temperature anomalies to identify the occurrence of dengue epidemics in Yogyakarta, Indonesia and Bangkok, Thailand [26]. Lowe et al. developed an epidemic early warning system for Southeast Brazil using several climate and non-climate datasets [27]. More recently, Huang et al. found that El Nino-Southern Oscillation climate cycles and temperature were important factors affecting the weekly occurrence of the four dengue serotypes in Cairns, Australia [23]. Adde et al. also identified summer Equatorial Pacific Ocean sea surface temperatures and Azore high sea-level pressure as significant indicators in predicting dengue epidemics in French Guiana [28]. While some of the climate factors were more commonly used due to the nature of a vector-borne disease, their applications varied and were geographically focused. These findings from previous literature showed that climate factors play a significant role in the occurrence of dengue epidemics.

This study first attempts to predict a dengue epidemic by developing an Early Warning Signal (EWS) model based upon the temporal relationship between the occurrence of dengue epidemics and climate variability which affects mosquito populations in Colombia. Furthermore, using climate data and topographical information, the study identifies population at high risk for dengue fever for efficient disease prevention activities.

## Methods

Dengue Incidence Proxy (DIP) was created to observe the trend of the dengue incidence in Colombia. The number of dengue fever cases and population data were obtained from SIVIGILA and Departamento Administrativo Nacional de Estadistica (DANE) which are both official governmental programs in Colombia [4, 29]. Dividing the dengue fever cases reported by population can be used as a good proxy to observe the overall trend of dengue fever. SIVIGILA also provides a weekly report on epidemiological events (Boletin Epidemiologico) which discloses the proportions of municipalities that were not responsive for each department [30]. Thus, the number of cases was adjusted by the proportions for underreporting by assuming that a non-responsive municipality would have the average number of cases per responsive municipality of that department: the reported cases by department was divided by the number of the responsive municipalities in that department, applied to non-responsive municipalities, and added to the reported cases by department. DIP was estimated by dividing the adjusted cases by population. While Boletin Epidemiologico was available over the study period, a more consistent pattern of the underreporting system was observed in the reports since 2011 after the large outbreak in 2010. Because a robust case reporting system is critical for determining the relationships between DIP and climate data, some departments out of 31 departments were excluded if over 20% of underreporting based on Boletin Epidemiologico occurred more than twice since 2011. An outbreak was defined as a relative term in this study. In other words, as long as an unusual peak in DIP was observed in a department, it was considered as an outbreak even if the DIP value in that department was relatively low compared to other departments where dengue is more prevalent. An unusual peak was marked by department if the slope of DIP over every six months fell into the highest 10% of the observations.

Table 1 summarized the datasets used in this study. Considering the spatial and temporal dynamics of mosquito populations, three climate datasets and two non-climate datasets were selected as factors which can explain variation in DIP. The climate raster datasets include air temperature, precipitation, and specific humidity [31–33]. The monthly climate datasets were obtained from 2006 to 2015, and all the raster files were resampled into 0.008 by 0.008 degree resolution by taking the nearest neighbor assignments. It should be noted that the study presumed that it is critical to consider how long favorable conditions for vector mosquitoes persist [9, 23]. In other words, a current epidemic is a result of the climate conditions consistently observed during the past months, rather than single temporal (monthly or daily) values at present. For example, if warm temperature and high humidity were observed only for a short time period of each year, these conditions would less likely affect the larval development or virus replication to cause an epidemic [25]. Thus, after checking cross-correlograms to define a proper period, the 12-month moving average of the mean values of each climate data was estimated by department (Additional file 1).

**Table 1:** Data description

Type	Degree resolution	Resampled resolution^a^	Temporal resolution	Period	Period (12MA)^b^
Air temperature	0.5 by 0.5	0.008 by 0.008	Monthly	Jan 2006 - Dec 2015	Jan 2007 - Dec 2015
Precipitation	1 by 1	0.008 by 0.008	Monthly	Jan 2006 - Dec 2015	Jan 2007 - Dec 2015
Specific humidity	2.5 by 2.5	0.008 by 0.008	Monthly	Jan 2006 - Dec 2015	Jan 2007 - Dec 2015
Night lights	0.5 by 0.5	0.008 by 0.008	Yearly	2006–2013	2007–2013
Elevation	0.5 by 0.5	0.008 by 0.008	NA	NA	NA

^a^Climate datasets were resampled by using the nearest option in ArcGIS

^b^12-month moving average

In addition to the climate factors, night light data and elevation raster files were included [34, 35]. Night lights data which is available by year was used to understand population density instead of conventional population statistics. The use of the night-lights data provides more flexibility to estimate population density at various levels of geographical units over time than the projected population data [36]. Prior to applying the night-lights data, correlations between night-lights data and population data were tested to ensure that the night-lights data can be used as an appropriate proxy (ρ = 0.94). The most recent night-lights data was for 2013 at the time of the research. As the population level does not change dramatically during a short period of time, the population level in 2013 was assumed to be consistent in 2014 and 2015. High population density would have two opposite effects in terms of transmission intensity depending upon the level of a reproduction number: (1) dilution of infectious individuals by having a large pool of host populations, (2) a large number of susceptible hosts to be infected, leading to the surge of infected cases. For the latter case, while transmission would be more intensive in a place where population density is high, holding other climate factors constant, it does not have to be necessarily true in areas at high elevations [9]. A previous study found that it is difficult for *Aedes aegypti* mosquitoes to survive at an elevation of 6000–8000 ft or even at lower elevations in temperate latitudes [37]. Because many people in Colombia live at high elevations (i.e. Bogota), the mean value of the night lights was used to estimate population density separately for people living under 1500 m and those living over 1500 m by department [38].

The three climate datasets are partially correlated but also have their own distinctive characteristics. In order to preserve all the information contained in each of the climate datasets, the Climate Risk Factor (CRF) index was created. The advantage of using a composite index is that it prevents multicollinearity when running regressions against independent variables with some level of the correlations among the variables. The three climate variables and population density under 1500 m were used by department. The precipitation variable, which has a negative relationship with DIP, was reversed, so all variables go towards the same underlying concept (the increase in DIP). The variables were first standardized individually by subtracting the mean and dividing by the standard deviation. The standardized values were then averaged across the variables [36, 39]. The final values were converted into a range from zero (low risk) to one (high risk) and multiplied by 100 for an easier interpretation. It should be noted that the temperature and specific humidity data used in this study are measures at the surface level. More precisely, air temperature is at 2 m above the ground surface, and specific humidity is measured near surface at sea level with pressure level 1000 millibars. Thus, it would be desirable to adjust the CRF index by the risk proportion at low and high elevation. The proportion at risk was estimated by dividing the sum of the night lights observed under 1500 m elevation by the sum of the total night lights in each department. The final CRF index was the product of the raw CRF index and the proportion at risk.

There were two dominant patterns observed during past dengue epidemics in Colombia: (1) rapid increase of the CRF index, (2) relatively steady increase of the CRF index at different levels of the CRF and DIP values. In other words, the slope of the CRF index curve at various levels of the CRF index and DIP values appeared to be critical in predicting the occurrence of dengue epidemics. In order to assess this combined relationship, the elasticity of the CRF index curve was estimated. This is defined as the percentage change in DIP in response to a 1 % change in the CRF index [40, 41]. The stationarity of the dataset was tested to ensure that there were no trend and periodic seasonal effects. The augmented Dickey-Fuller (ADF) unit-root test was used to test whether the dataset is stationary by department [42, 43]. DIP is non-negative integer values, and count models were used to fit DIP as a function of the CRF index (Additional file 1: Supplementary 2). The DIP dataset consists of two parts: (1) model dataset, (2) validation dataset. The model was constructed based on monthly DIP and the CRF index by department from January 2007 to December 2015. The validation dataset which was separated from the model dataset was established from January 2016 to April 2016 and used to validate the model performance. Overdispersion—where the variance is greater than the mean—was tested using the Z-score test at the 5% significant level [44–46]. In addition, the Akaike Information Criterion (AIC) fit test was used to compare the model fits between Poisson and negative binomial models. Being a non-linear model, the elasticity of the CRF function can be given as [46]:

$$ E lasticity=\frac{\partial E\left({y}_i|{x}_i\right)}{\partial {x}_i}\bullet \frac{x}{y}= \exp \left({x}_i^{\hbox{'}}{\beta}_k\right){\beta}_k\bullet \frac{x}{y} $$

where $\exp \left({x}_i^{\prime}\beta \right)$ is the expected DIP values, *β*_*k*_ is the coefficient of CRF, *x* is the explanatory, and *y* is the response.

As shown above, the main interest of the study lay in estimating elasticities, and count models were used as an intermediary step in calculating elasticities. Given the geographical variations of dengue outbreaks, it is critical to estimate the elasticities separately by department with varying coefficient values of CRF. In this context, the current model was preferred to non-linear mixed models with a fixed coefficient and random effects since the use of coefficients and the measure of marginal effects and elasticities were more straightforward, reducing any possibility of potential overspecification (i.e. multiple adjustments) [46, 47]. Because the model was run separately for each department allowing variation in the CRF index by department, there is no concern about creating the effect of spatial autocorrelation. The elasticities were derived for every six months from January 2007 to December 2015. Early Warning Signal (EWS) was modeled such that dengue epidemics in Colombia can likely occur when the elasticity of the CRF index is maximized given the instantaneous slopes of DIP and the CRF index over time are positive minimizing the squared residuals.

Maximize:

$$ Elasticity, E $$

Subject to:

$$ (1)\ {\beta}_{DIP}=\frac{\sum_{i= t}^{t+5}\left({DIP}_i-\overset{-}{DIP}\right)}{\sum_{i= t}^{t+5}\left({T}_i-\overset{-}{T}\right)}>0 $$

and

$$ (2)\ {\beta}_{CRF}=\frac{\sum_{i= t}^{t+5}\left({CRF}_i-\overset{-}{CRF}\right)}{\sum_{i= t}^{t+5}\left({T}_i-\overset{-}{T}\right)}>0 $$

where $\overset{-}{DIP}\  and\ \overset{-}{CRF}$ are the means of *DIP and CRF*, T is time (month). The elasticities were then categorized into three percentiles: low level warning (0–50%), medium level warning (50–75%), and high level warning (75–100%). As expressed by Adde et al., the hit rate (HR) and false alarm rate (FAR) were defined as below [28]:

$$ HR\ \left( or\  sensitivity\right)=\frac{\left( Detections\ |\  Outbreak\right)}{\left( Detections+ Misses\ |\  Outbreak\right)} $$

$$ FAR\ \left( or\ 1- specificity\right)=\frac{\left( False\  signals\ |\  No\  outbreak\right)}{\left( No\  signals+ False\  signals\ |\  No\  outbreak\right)} $$

In addition, a sensitivity analysis was conducted with various moving average scenarios to make sure that the 12-month moving average is the most suitable period for the performance of the EWS model.

Given that the CRF index is statistically significant to explain variance of DIP for the departments where significant underreporting was not observed, the CRF index was further estimated at the smaller geographical level (5 km by 5 km resolution) for the entire country and used to identify high risk areas.

## Results

**Fig. 1:**
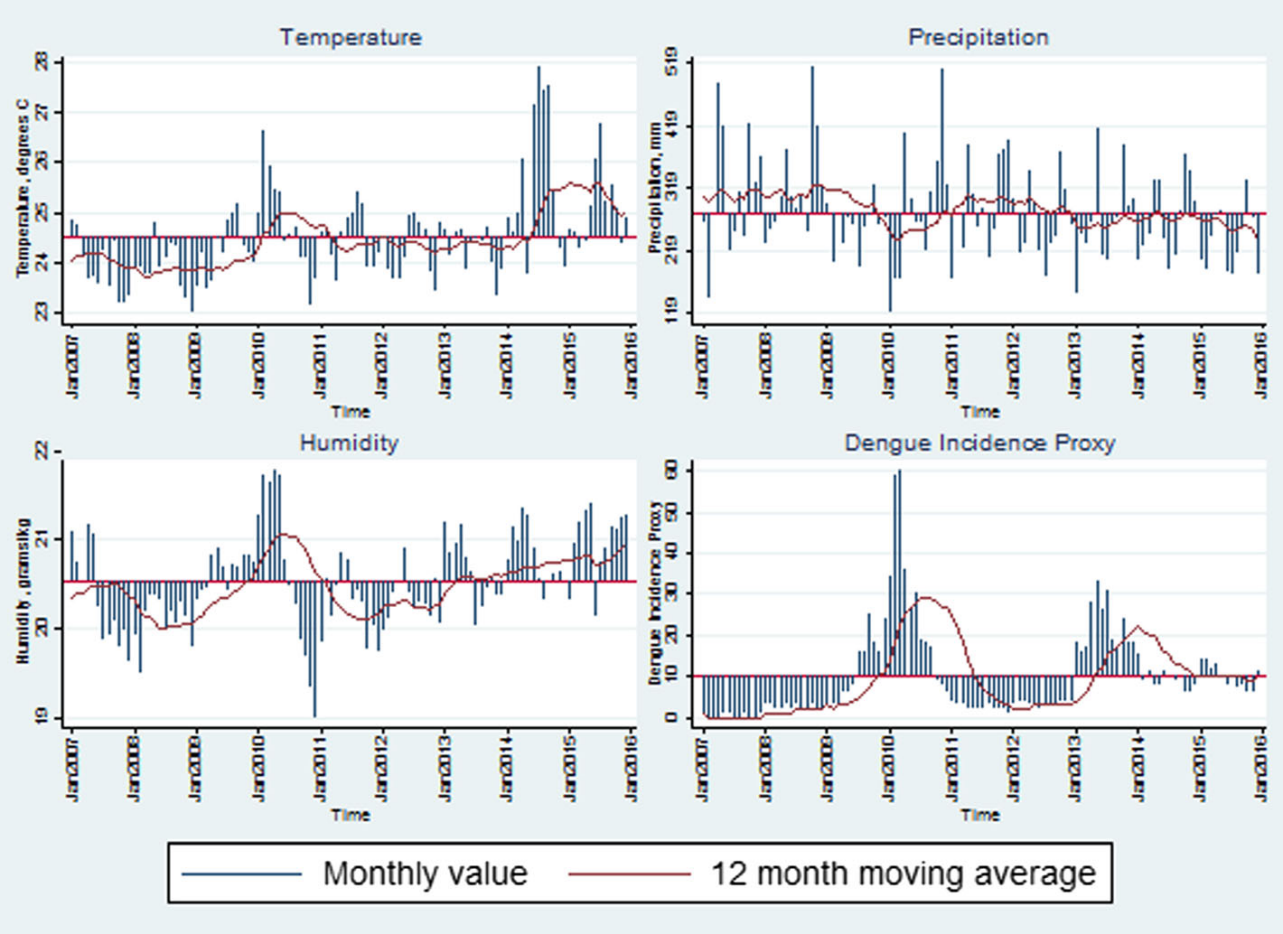
Climate factors and DIP over time in Valle del Cauca^*^. ^*^ See Additional file 1: Supplementary 3 for other departments

During the period from January 2007 to December 2015, two major outbreaks were observed in many parts of Colombia. Figure 1 presents the overall trends of the three climate factors, as well as the DIP from 2007 to 2015 in Valle del Cauca, one of the departments where dengue fever is highly prevalent (see Additional file 1: Supplementary 3 for other departments). Looking at the bottom right panel in Fig. 1, there were two major outbreaks in 2010 and 2013 in the department. Comparing the trend of DIP with the climate factors, DIP appears to be positively correlated with temperature and humidity, but has a negative relationship with precipitation.

13 of 31 departments in Colombia were chosen after checking the robustness of the case reporting system. The ADF test showed that we reject the null hypothesis, which means that the dataset is stationary. As shown in Table 2, the CRF index is highly significant for all departments except Guaviare and Magdalena, thus 11 departments were selected for further analysis.

**Table 2:** Regression outputs of the CRF index on DIP

Department	Number	Selected model	*P*-value for Z-score test (overdispersion)	Constant (α)		CRF (β)		AIC^b^	AIC (comparison)^c^
Antioquia	108	Poisson	0.47	−2.89	***	0.31	***	3.84	4.26
Arauca	108	NB^a^	0.00	2.00	***	0.06	***	8.46	30.37
Boyaca	108	NB	0.05	−0.59		0.51	***	4.26	4.19
Cauca	108	Poisson	0.19	−0.45	*	0.08	***	3.53	3.58
Cundinamarca	108	NB	0.03	−5.35	***	0.52	***	2.55	2.26
Guaviare	108	NB	0.02	3.23	***	−0.01		7.69	16.72
Huila	108	NB	0.00	0.49		0.05	***	7.87	14.33
Magdalena	108	NB	0.00	0.92		0.02		5.44	7.05
Norte de Santander	108	NB	0.00	1.61	***	0.05	**	7.68	10.54
Quindio	108	NB	0.02	−3.01	***	0.11	***	7.41	21.83
Risaralda	108	Poisson	0.11	−0.62	*	0.07	***	4.56	4.69
Santander	108	NB	0.00	1.07	*	0.06	**	7.12	9.89
Valle del Cauca	108	NB	0.00	−2.75	***	0.12	***	6.20	8.68

^a^Negative Binomial

^b^Akiake Information Criterion

^c^AICs for non-selected count models were presented for comparison. The AIC fit test was consistent with the Z-score test in terms of choosing a better model fit except Boyaca and Cundinamarca. Since the AIC differences were trivial for the two departments, the Bayesian Information Criterion (BIC) was further assessed, and NB was preferred over Poisson

* Significance at the 10% level, ** at the 5% level, *** at the 1% level

**Fig. 2:**
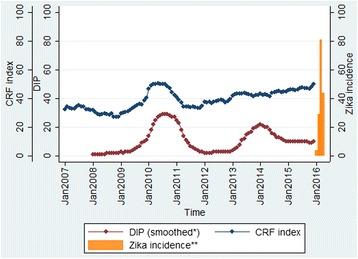
The CRF index and DIP over time in Valle del Cauca^***^. ^*^ DIP was smoothed out to reduce short-term fluctuations and highlight longer term trends for demonstration. ^**^Zika cases were reported in 2015 as well, but zika incidence rates (/100,000) were not clearly shown for year 2015 due to the low number of reported cases. ^***^ See Additional file 1: Supplementary 4 for other departments

**Fig. 3:**
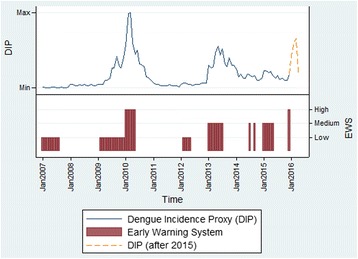
Early Warning Signal in Valle del Cauca

The CRF index and DIP were plotted over time to show the overall trend in Fig. 2 (see Additional file 1: Supplementary 4 for other departments). It is clear that the epidemic that occurred in 2010 was picked up by the steep increase of the CRF index. In 2013, another epidemic was observed. While there was no rapid change in terms of the CRF index during a short period in 2013, the CRF index reached its high level following the steady increase of the index since 2012. These provide an important point where an occurrence of future dengue epidemic can be related not only to the rapid increase of the CRF index, but also to the various levels of the CRF index and DIP. These combined relationships can be further explained by the elasticity of the CRF index which was used to develop an Early Warning Signal (EWS) model. In Fig. 3, the EWS based on the elasticity of the function was demonstrated for Valle del Cauca. In the department, the peak DIP was observed in March 2010, and the EWS signaled the high level warning sign two months before the peak (January 2010). Similarly, the second peak occurred in May 2013, and the EWS level went up from low to medium in January 2013 and remained at the same level until the end of the peak. It should be noted that there was no major outbreak observed throughout 2015 despite the continuous increase of the CRF index. Instead, Zika, another viral disease caused by *Aedes aegypti* emerged in 2015 and continued to increase in 2016. Overall, all 11 departments experienced dengue epidemics in 2010, and nine of them had additional minor outbreaks since 2011. Among the total of 24 observed outbreaks, EWS successfully detected 18 (75%) 1 ~ 5 months ahead of time and, three (12.5%) in the same month, and missed three (12.5%) (Additional file 1: Supplementary 6).

**Fig. 4:**
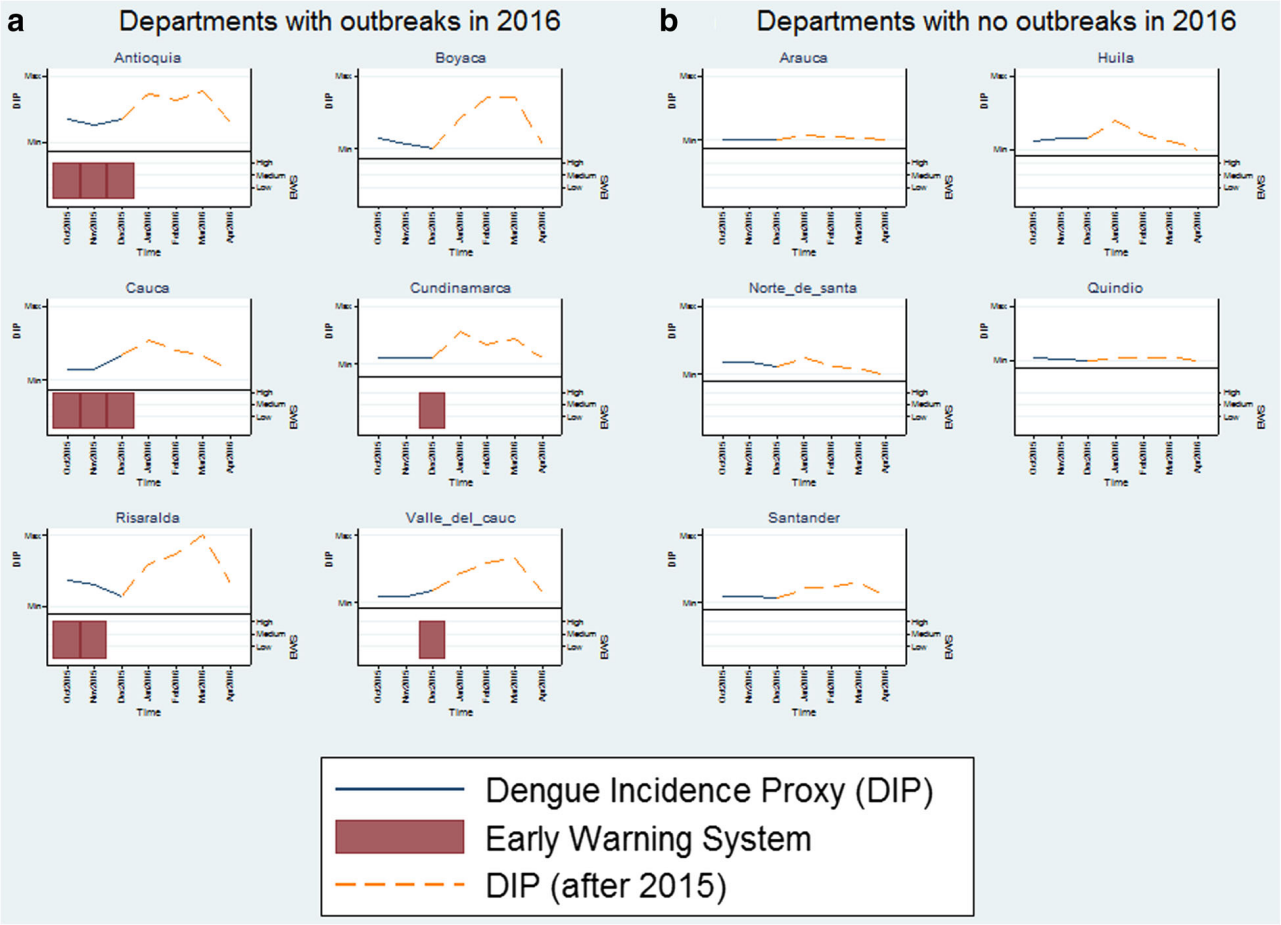
EWS accuracy with the validation data in 2016

The EWS model predictability was examined with the validation data in 2016 which was separated from the model. It is interesting to see that the EWS already signaled the high level warning sign at the end of 2015, which accurately predicted another outbreak in two months (February 2016) that is out of the study period. Figure 4 further demonstrates the EWS model performance with the validation data for all 11 departments. 6 of 11 departments experienced outbreaks between January 2016 and April 2016. The EWS model successfully predicted these outbreaks 1 ~ 5 months ahead of time for all departments except Boyaca (HR = 83.3%). In addition, the EWS model did not send out any false alarms for the other 5 departments where no outbreak occurred during the out-of-sample period (FAR = 0%). In other words, sensitivity (HR), specificity, positive predictive value, and negative predictive value of the validation data were as follows: 83.3%, 100%, 100%, and 83.3%.

**Fig. 5:**
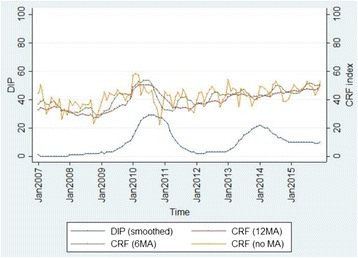
The CRF index with different moving average scenarios in Valle del Cauca

The sensitivity analysis was performed with different moving average scenarios (12 months, 6 months, current value). As shown in Table 3, the hit rate was the highest with the 12-month moving average scenario, meaning that the current model produced the most accurate prediction compared to the 6-month and no-moving-average scenarios. The false alarm rate increased as the moving average period was shortened. This is mainly because the index becomes too sensitive and changes quickly due to the short duration of the moving averages of the climate datasets. As a result, it does not distinguish between minor fluctuations and major outbreaks (Fig. 5). This sensitive behavior of the CRF index with the shorter term scenarios proves our presumption that a current dengue epidemic is a result of the consistent long-term patterns of the climate conditions.

**Table 3:** Sensitivity analysis with additional moving average scenarios

Scenario	HR (sensitivity)	FAR	Specificity	Positive Predictive Value	Negative Predictive Value
12 month MA	87.5%	3.1%	96.9%	91.3%	95.4%
6 month MA	75.0%	4.7%	95.3%	85.7%	91.0%
Current value (no MA)	83.3%	6.3%	93.8%	83.3%	93.8%

**Fig. 6:**
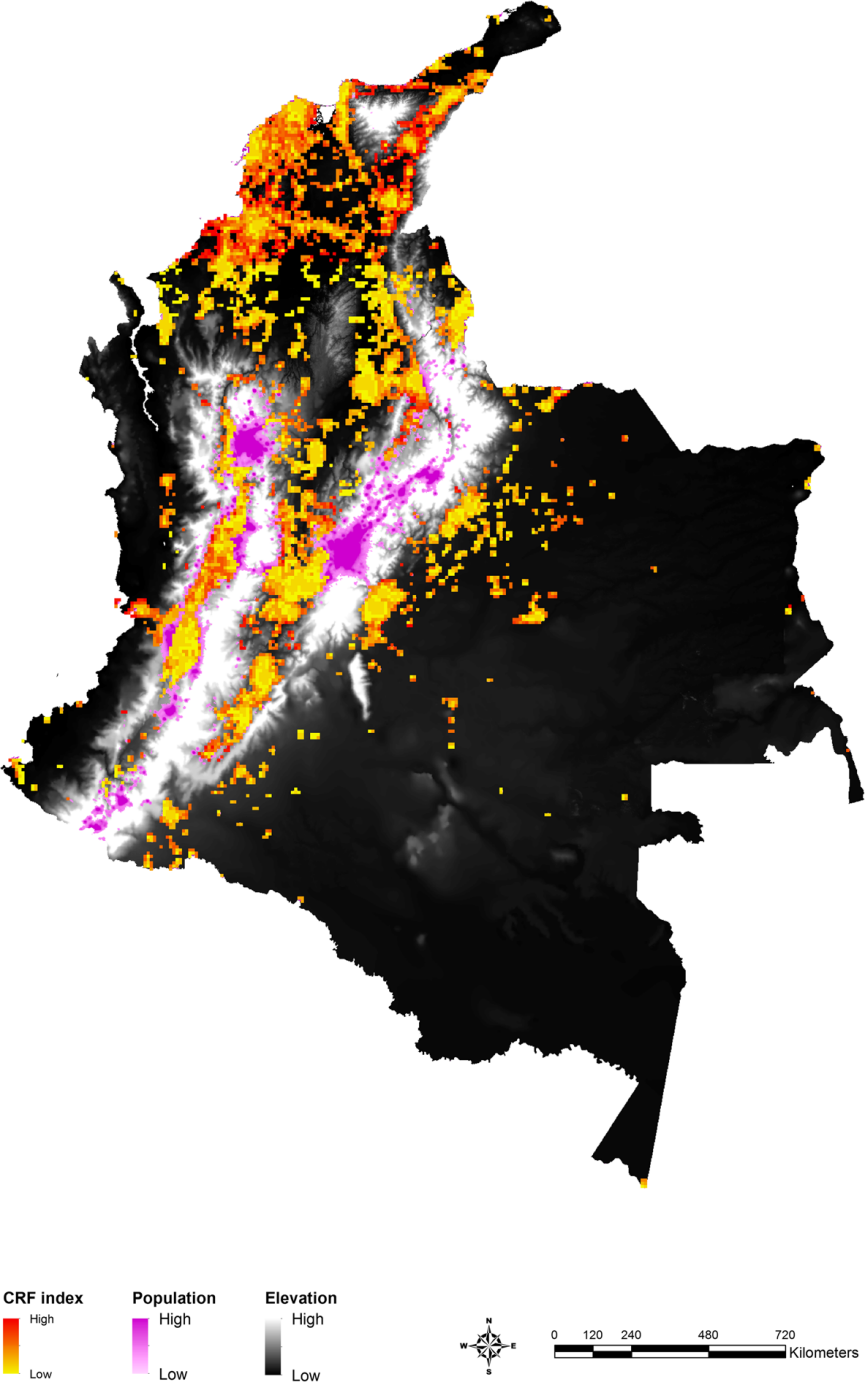
Identification of high risk areas in Dec, 2015^*^. ^*^ See Additional file 1: Supplementary 5 for more details

Given that the CRF index explains variation in DIP reasonably well, the CRF index was estimated at 5 km by 5 km resolution, and the most recent time of the index (December 2015) was presented in Fig. 6 (see Additional file 1: Supplementary 5 for more details). As expected, populations at high risk are concentrated in the Western part of the country due to more suitable climate conditions for vector mosquitoes and the high population level compared to the East. Using the geo-coordinates of the high risk areas at 5 km by 5 km resolution, it is possible to identify the locations for people at high risk more accurately for efficient disease prevention activities.

## Discussion

This study confirms that dengue fever transmission is strongly related to climate factors as well as population density at different topographical conditions. One of the advantages of the CRF index is to prevent multicollinearity by combining all relevant climate indicators which may have some degrees of correlations with each other but have distinctive characteristics at the same time. During the study period from January 2007 to December 2015, the nationwide dengue epidemic occurred in 2010 was well explained by the rapid changes of the CRF index. Even if the CRF index increased steadily, the study found that it was still possible to detect an epidemic by adopting the elasticity of the function which takes into account not only the slopes but also the various levels of CRF and DIP.

In 2015, some inconsistent patterns between CRF and DIP were observed for some departments (Additional file 1: Supplementary 7). This inconsistency may be related to the unexpected emergence of Zika, which started being reported in 2015. As shown in Fig. 2, the number of Zika cases has continuously increased since 2015. However, it is still premature to make any firm statements regarding the impact of Zika on dengue fever due to uncertainty of the diseases. Given that reported cases are mainly based on clinical symptoms, there may have been a chance of misdiagnosis between the two diseases. In addition, due to the surge of an unfamiliar disease (Zika) imposing more difficulties on resource allocation at the local health facility level, it would be difficult to keep a consistent pattern in the case-reporting system from municipality-level heath facilities. Excluding 2015, a number of false alarms where EWS sends out the medium or high level signals but DIP remains low were only observed twice in Cauca (April and December 2014) during the study period.

Some areas of uncertainty deserve attention. While the CRF index performed well for 11 of 13 departments, the index was not statistically significant in Magdalena and Guaviare. This may have been caused partly by the inconsistent patterns of reported cases over time. Because the EWS was estimated based upon the most recent observed climate datasets, the EWS in this study is limited to issuing alerts with short-time intervals (1 ~ 5 months ahead). Given that, at present there are 1 ~ 2 month delays until the climate data become available, EWS with the short intervals (i.e. less than two months) may not, for now, be practical in operational modes. However, this limitation can be improved based upon the availability of the climate datasets in real-time in the future, and the 1 ~ 5 month intervals would provide enough room for public health officials to prepare for selected vector control activities and healthcare interventions (i.e. increase the number of beds at high risk areas) in the dengue-endemic setting [9, 26]. It should be noted that the study did not attempt to produce any longer-term predictions due to chaos and uncertainty in climate forecasts in the long run. Considering that long-term climate forecasts could be variable depending upon assumptions (i.e. future CO_2_ omission level), the method proposed in this study could minimize potential bias which may be caused by uncertainty in input datasets. The climate datasets have coarse resolutions. While the datasets were resampled using the nearest option in this study, the model outcomes can be further improved with finer scale resolutions. It is worth noting that the cycling of El Niño and La Niña, called El Niño Southern Oscillation (ENSO), may have indirect impacts on the occurrence of dengue epidemics in South America by changing the patterns of climate variables such as temperature, precipitation, and humidity [28]. While any unusual changes of the climate variables affected by such events were captured by using the 12-month moving averages, further investigation would be needed to identify accurate impacts of El Niño on climate factors including its timing.

Nonetheless, our model provided accurate forecasts for the validation period for 5 of 6 departments that experienced outbreaks in 2016. In addition, this study identified populations at high risk for dengue at 5 km by 5 km resolution. The study findings can be used to accelerate introduction of dengue prevention activities and prioritize alternative health interventions among competing health demands in Colombia.

## Conclusions

The CRF index summarized multiple climate and non-climate risk factors into a single indicator which helps decision makers to understand easily [36]. While some of the climate factors were more commonly used in the existing literature due to the nature of a vector-borne disease, the applications of the climate data in these studies appeared to vary. The proposed EWS model in this study used the concept of elasticity to understand how DIP changes to varying levels of the CRF index and successfully detected dengue outbreaks in Colombia. In addition, the CRF index was further estimated at 5 km by 5 km resolution. The areas where the CRF index values have been continuously high over time can be prioritized for appropriate healthcare interventions. Furthermore, this can guide decision makers to find relevant locations where future surveillance studies can be conducted.

## Additional files

Additional file 1: Supplementary 1.Cross-correlograms of climate datasets and DIP. Supplementary 2. Model specifications. Supplementary 3. Climate factors and DIP over time by department. Supplementary 4. The CRF index and DIP over time by department. Supplementary 5. Identification of high risk areas for dengue fever. Supplementary 6. EWS for 11 departments during the study period. Supplementary 7. EWS for 11 departments in 2015. (ZIP 2317 kb)

## Abbreviations

ADE: Antibody Dependent Enhancement
ADF: Augmented Dickey Fuller
AIC: Akaike Information Criterion
CRF: Climate Risk Factors
DIP: Dengue Incidence Proxy
EWS: Early Warning Signal
