# Supplementary material for: Early warning signal for dengue outbreaks and identification of high risk areas for dengue fever in Colombia using climate and non-climate datasets
Source: BMC Infect Dis. 2017 Jul 10;17:480. doi: 10.1186/s12879-017-2577-4 (PMC5504639; doi:10.1186/s12879-017-2577-4)
Supplement: Supplementary file 1 — Supplementary 1.Cross-correlograms of climate datasets and DIP. Supplementary 2. Model specifications. Supplementary 3. Climate factors and DIP over time by department. Supplementary 4. The CRF index and DIP over time by department. Supplementary 5. Identification of high risk areas for dengue fever. Supplementary 6. EWS for 11 departments during the study period. Supplementary 7. EWS for 11 departments in 2015. (ZIP 2317 kb) [file 12879_2017_2577_MOESM1_ESM.zip › Supplementary_v11R2.docx]

**Supplementary 1. Cross-correlograms of climate datasets and DIP**

A cross-correlogram is useful to check correlations between one variable and another variable with time lags. This study presumed that a current epidemic is a result of the climate conditions observed during the past months, rather than single temporal values at present. In this regard, cross-correlations between various periods (current, 6, 12 months) of the moving averages of the climate datasets and observed Dengue Incidence Proxy (DIP) with time lags were examined. The 12-month moving average was selected as the most appropriate period which explains the current status (lag 0) of DIP. In the figure below, the 12-month moving averages were plotted against DIP with time lags. Overall, while temperature and humidity are positively related to DIP at lag 0, precipitation shows negative relationships with the current status of DIP. It should be noted that more consistent and stronger correlations were observed for precipitation and humidity than temperature across departments.

**Supplementary Figure 1(a). Cross-correlation with temperature**

**Supplementary Figure 1(b). Cross-correlation with precipitation**

**Supplementary Figure 1(c). Cross-correlation with humidity**

**Supplementary 2. Model specifications (Hilbe 2011, Haab and McConnel 2002)**

The Poisson probability density function can be written as:

$$\Pr\left( y_{i}=n \right)=\frac{e^{-\lambda_{i}}\lambda_{i}^{n}}{n!}, n=0, 1, 2\ldots$$

where *n* is observed count, and $\lambda_{i}$ is the mean, $\lambda_{i}=exp(x_{i}\beta)$. For the Poisson model, the mean is equal to the variance of the distribution. However, the variance tends to be greater than the mean in many situations, and this is called overdispersion. When overdispersion exists, the standard errors estimated in the Poisson mode are underestimated, causing the rejection of null hypotheses of no association.

The negative binomial technique relaxes the assumption of equality of the mean and variance by adding a gamma distributed error term. A common version of the negative binomial model is as follows:

$$E\left( y_{i} | x_{i}\beta\right)=\lambda_{i}=\exp\left( x_{i}\beta\right)$$

$$\log\left( E\left( y_{i} \right) \right)=x_{i}\beta+\theta_{i}$$

where $\theta_{i}$ represents unobserved individual differences (or unobserved heterogeneity).

$$\Pr\left( y \right)=\frac{\Gamma(y_{i}+\frac{1}{\alpha})}{\Gamma\left( y_{i}+\frac{1}{\alpha} \right)\Gamma(\frac{1}{\alpha})}\left( \frac{\frac{1}{\alpha}}{\frac{1}{\alpha}+\lambda_{i}} \right)^{\frac{1}{\alpha}}\left( \frac{\lambda_{i}}{\frac{1}{\alpha}+\lambda_{i}} \right)^{y_{i}}$$

where $\lambda_{i}=\exp\left( x_{i}\beta\right)$. The mean of the negative binomial distribution is ${E\left( y_{i} \right)=\lambda}_{i}=\exp\left( x_{i}\beta\right)$. However, now the variance of the dependent variable is ${V\left( y_{i} \right)=\lambda}_{i}(1+\alpha\lambda_{i})$. The parameter $\alpha$ is considered to be the overdispersion parameter.

In order to check overdispersion, Z-tests score was used. Z-tests score examines the hypothesis that the Poisson model is overdispersed.

$$Z_{i}=\frac{\left( y_{i}-\mu_{i} \right)^{2}-y_{i}}{\mu_{i}\sqrt{2}}$$

where $y_{i}$ = observation i, $\mu_{i}$ = predicted value of i.

After regressing Z, if Z coefficient is significant, the hypothesis of no overdispersion is rejected.

**Supplementary 3. Climate factors and DIP over time by department**

**Supplementary Figure 3(a). Antioquia**

**Supplementary Figure 3(b). Arauca**

**Supplementary Figure 3(c). Boyaca**

**Supplementary Figure 3(d). Cauca**

**Supplementary Figure 3(e). Cundinamarca**

**Supplementary Figure 3(f). Huila**

**Supplementary Figure 3(g). Norte de Santa**

**Supplementary Figure 3(h). Quindio**

**Supplementary Figure 3(i). Risaralda**

**Supplementary Figure 3(j). Santander**

**Supplementary 4. The CRF index and DIP over time by department**

**Supplementary Figure 4**

* DIP was smoothed out to reduce short-term fluctuations and highlight longer term trends for demonstration

**Zika cases were reported in 2015 as well, but zika incidence rates (/100,000) were not clearly shown for year 2015 due to the low number of reported cases

**Supplementary 5. Identification of high risk areas for dengue fever**

Given that the CRF index explains variation in DIP reasonably well, the CRF index was estimated at 5km by 5km resolution, and the most recent time of the index (December 2015) was presented in Supplementary figure 5. Figure 5(a) depicts the CRF index without taking into account the population at different elevation levels. Overall, the Western part of Colombia is at higher risk than the Eastern part of the country. Apart from the impact of the climate factors, this is also partly due to population density. Looking at Figure 5(b), population density appeared to be a lot higher in the North-Western areas of Colombia compared to the South-Eastern. The CRF index was then adjusted by the risk proportion and shown in Figure 5(c). As expected, populations at high risk are concentrated in the Western part of the country due to more suitable climate conditions for vector mosquitoes and the high population level compared to the East. In fact, the total number of cases reported from four departments in the East side of the country (Amazonas, Guainia, Vaupes, and Vichada) was only 2.5% of the total cases (5,524) reported from Valle del Cauca alone throughout 2015. There are areas where high population level was observed (purple in Figure 5(c)) but not identified as high-risk areas. This is the population living at high elevation. Bogota would be a good example where dengue has not been prevalent despite the high level of population density in the city. Lastly, Figure 5(d) shows the reported dengue cases in December 2015 by municipality. It is clear to see that the high risk areas identified by the model (Figure 5(c)) are consistent with the municipalities with the actual high caseloads shown in Figure 5(d). Using the geo-coordinates of the high risk areas at 5km by 5km resolution, it is possible to identify the locations for people at high risk more accurately for the disease prevention activities.

**Supplementary Figure 5. CRF index before and after the adjustment of population at low elevation in Dec, 2015**

**Supplementary 6. EWS for 11 departments during the study period**

The table below shows Early Warning Signals (EWS) during the study period for 11 departments. 23 outbreaks were detected using the slope of DIP, and one unusual peak was manually added after qualitative adjustment. Correct warning signals 1~5 months ahead of time, correct warning signals in the same month, and missed warning signals were marked as a, b, and c, respectively. In addition, sensitivity (SE), specificity (SP), positive predictive value (PPV), and negative predictive value (NPV) were summarized in Table S6(b).

Table S6(a)

| **Department** | **Time** | **DIP^d^** | **Outbreak peak** | **EWS^e^** |
| --- | --- | --- | --- | --- |
| Antioquia | Nov-09 | 3 |  | Low |
| Antioquia | Dec-09 | 5 |  | Low |
| Antioquia^a^ | Jan-10 | 4 |  | High |
| Antioquia | Feb-10 | 7 |  | High |
| Antioquia | Mar-10 | 11 |  | High |
| Antioquia | Apr-10 | 12 |  | High |
| Antioquia | May-10 | 19 |  | High |
| Antioquia | Jun-10 | 23 | V | High |
| Antioquia | Jul-10 | 15 |  | . |
| Antioquia | Aug-10 | 10 |  | . |
| … | … | … |  | … |
| Antioquia | May-15 | 2 |  | . |
| Antioquia | Jun-15 | 3 |  | . |
| Antioquia^a^ | Jul-15 | 6 |  | High |
| Antioquia | Aug-15 | 4 |  | High |
| Antioquia | Sep-15 | 9 | V | High |
| Antioquia | Oct-15 | 8 |  | High |
| Antioquia | Nov-15 | 6 |  | High |
| Antioquia | Dec-15 | 8 |  | High |
|  |  |  |  |  |
| **Department** | **Time** | **DIP** | **Outbreak peak** | **EWS** |
| Arauca | Apr-09 | 21 |  | . |
| Arauca^a^ | May-09 | 50 |  | Low |
| Arauca | Jun-09 | 52 |  | Low |
| Arauca | Jul-09 | 77 | V | Low |
| Arauca | Aug-09 | 70 |  | Low |
| Arauca | Sep-09 | 55 |  | Low |
| Arauca | Oct-09 | 41 |  | . |
| … | … | … |  | … |
| Arauca | Jan-10 | 43 |  | . |
| Arauca^a^ | Feb-10 | 61 |  | High |
| Arauca | Mar-10 | 102 |  | High |
| Arauca | Apr-10 | 141 |  | High |
| Arauca | May-10 | 212 | V | High |
| Arauca | Jun-10 | 203 |  | High |
| Arauca | Jul-10 | 167 |  | High |
| Arauca | Aug-10 | 47 |  | . |
| Arauca | Sep-10 | 18 |  | . |
| … | … | … |  | … |
| Arauca | Mar-13 | 30 |  | . |
| Arauca | Apr-13 | 35 |  | . |
| Arauca^a^ | May-13 | 51 |  | Low |
| Arauca | Jun-13 | 34 |  | Low |
| Arauca | Jul-13 | 67 | V^*^ | Low |
| Arauca | Aug-13 | 27 |  | Low |
| Arauca | Sep-13 | 33 |  | . |
| Arauca | Oct-13 | 39 |  | . |
|  |  |  |  |  |
| **Department** | **Time** | **DIP** | **Outbreak peak** | **EWS** |
| Boyaca | Nov-09 | 2 |  | . |
| Boyaca | Dec-09 | 3 |  | Low |
| Boyaca^a^ | Jan-10 | 4 |  | Medium |
| Boyaca | Feb-10 | 11 |  | Medium |
| Boyaca | Mar-10 | 5 |  | Medium |
| Boyaca | Apr-10 | 8 |  | Medium |
| Boyaca | May-10 | 14 | V | Medium |
| Boyaca | Jun-10 | 12 |  | Medium |
| Boyaca | Jul-10 | 7 |  | . |
| Boyaca | Aug-10 | 7 |  | . |
|  |  |  |  |  |
| **Department** | **Time** | **DIP** | **Outbreak peak** | **EWS** |
| Cauca | Nov-09 | 2 |  | Low |
| Cauca | Dec-09 | 7 |  | Low |
| Cauca^a^ | Jan-10 | 10 |  | Medium |
| Cauca | Feb-10 | 15 | V | Medium |
| Cauca | Mar-10 | 8 |  | Medium |
| Cauca | Apr-10 | 4 |  | Medium |
| Cauca | May-10 | 3 |  | . |
| Cauca | Jun-10 | 2 |  | . |
| … | … | … |  | … |
| Cauca | Jul-15 | 1 |  | . |
| Cauca | Aug-15 | 1 |  | . |
| Cauca^a^ | Sep-15 | 1 |  | High |
| Cauca | Oct-15 | 2 |  | High |
| Cauca | Nov-15 | 2 |  | High |
| Cauca | Dec-15 | 5 | V | High |
|  |  |  |  |  |
| **Department** | **Time** | **DIP** | **Outbreak peak** | **EWS** |
| Cundinamarca | Nov-09 | 1 |  | Low |
| Cundinamarca | Dec-09 | 2 |  | Low |
| Cundinamarca^a^ | Jan-10 | 3 |  | High |
| Cundinamarca | Feb-10 | 2 |  | High |
| Cundinamarca | Mar-10 | 9 | V | High |
| Cundinamarca | Apr-10 | 4 |  | High |
| Cundinamarca | May-10 | 4 |  | High |
| Cundinamarca | Jun-10 | 3 |  | . |
| Cundinamarca | Jul-10 | 1 |  | . |
| … | … | … |  | … |
| Cundinamarca | Nov-12 | 1 |  | Low |
| Cundinamarca | Dec-12 | 1 |  | Low |
| Cundinamarca^a^ | Jan-13 | 2 |  | Medium |
| Cundinamarca | Feb-13 | 2 |  | Medium |
| Cundinamarca | Mar-13 | 2 |  | Medium |
| Cundinamarca | Apr-13 | 2 |  | Medium |
| Cundinamarca | May-13 | 4 | V | Medium |
| Cundinamarca | Jun-13 | 2 |  | Medium |
| Cundinamarca | Jul-13 | 3 |  | . |
| Cundinamarca | Aug-13 | 2 |  | . |
|  |  |  |  |  |
| **Department** | **Time** | **DIP** | **Outbreak peak** | **EWS** |
| Huila | Nov-09 | 23 |  | Low |
| Huila | Dec-09 | 45 |  | Low |
| Huila^a^ | Jan-10 | 65 |  | High |
| Huila | Feb-10 | 75 | V | High |
| Huila | Mar-10 | 62 |  | High |
| Huila | Apr-10 | 56 |  | High |
| Huila | May-10 | 62 |  | High |
| Huila | Jun-10 | 64 |  | . |
| Huila | Jul-10 | 42 |  | . |
| … | … | … |  | … |
| Huila | Nov-11 | 14 |  | . |
| Huila | Dec-11 | 14 |  | . |
| Huila | Jan-12 | 28 |  | Low |
| Huila | Feb-12 | 45 |  | Low |
| Huila | Mar-12 | 38 |  | Low |
| Huila | Apr-12 | 47 |  | Low |
| Huila^c^ | May-12 | 53 | V | Low |
| Huila | Jun-12 | 45 |  | Low |
| Huila | Jul-12 | 35 |  | . |
| Huila | Aug-12 | 23 |  | . |
| … | … | … |  | … |
| Huila | Nov-13 | 18 |  | . |
| Huila | Dec-13 | 16 |  | . |
| Huila^a^ | Jan-14 | 51 |  | Medium |
| Huila | Feb-14 | 55 | V | Medium |
| Huila | Mar-14 | 52 |  | Medium |
| Huila | Apr-14 | 44 |  | . |
| Huila | May-14 | 33 |  | . |
|  |  |  |  |  |
| **Department** | **Time** | **DIP** | **Outbreak peak** | **EWS** |
| Norte de Santander | Nov-09 | 21 |  | Low |
| Norte de Santander | Dec-09 | 41 |  | Low |
| Norte de Santander^b^ | Jan-10 | 65 | V | High |
| Norte de Santander | Feb-10 | 58 |  | High |
| Norte de Santander | Mar-10 | 47 |  | High |
| Norte de Santander | Apr-10 | 30 |  | High |
| Norte de Santander | May-10 | 31 |  | . |
| Norte de Santander | Jun-10 | 37 |  | . |
| … | … | … |  | … |
| Norte de Santander | Feb-13 | 19 |  | . |
| Norte de Santander | Mar-13 | 18 |  | . |
| Norte de Santander | Apr-13 | 27 |  | Low |
| Norte de Santander | May-13 | 32 |  | Low |
| Norte de Santander | Jun-13 | 25 |  | Low |
| Norte de Santander^c^ | Jul-13 | 45 | V | Low |
| Norte de Santander | Aug-13 | 20 |  | Low |
| Norte de Santander | Sep-13 | 23 |  | . |
| Norte de Santander | Oct-13 | 29 |  | . |
|  |  |  |  |  |
| **Department** | **Time** | **DIP** | **Outbreak peak** | **EWS** |
| Quindio | Nov-09 | 12 |  | Low |
| Quindio | Dec-09 | 20 |  | Low |
| Quindio^a^ | Jan-10 | 54 |  | High |
| Quindio | Feb-10 | 184 |  | High |
| Quindio | Mar-10 | 321 | V | High |
| Quindio | Apr-10 | 123 |  | High |
| Quindio | May-10 | 125 |  | High |
| Quindio | Jun-10 | 77 |  | . |
| Quindio | Jul-10 | 43 |  | . |
| … | … | … |  | … |
| Quindio | Nov-14 | 9 |  | . |
| Quindio | Dec-14 | 2 |  | . |
| Quindio^a^ | Jan-15 | 28 |  | Medium |
| Quindio | Feb-15 | 30 |  | Medium |
| Quindio | Mar-15 | 33 |  | Medium |
| Quindio | Apr-15 | 59 | V | Medium |
| Quindio | May-15 | 42 |  | Medium |
| Quindio | Jun-15 | 38 |  | Medium |
| Quindio | Jul-15 | 46 |  | High |
| Quindio | Aug-15 | 22 |  | . |
| Quindio | Sep-15 | 26 |  | . |
|  |  |  |  |  |
| **Department** | **Time** | **DIP** | **Outbreak peak** | **EWS** |
| Risaralda | Nov-09 | 8 |  | Low |
| Risaralda^c^ | Dec-09 | 18 | V | Low |
| Risaralda | Jan-10 | 8 |  | High |
| Risaralda | Feb-10 | 8 |  | High |
| Risaralda | Mar-10 | 10 |  | High |
| Risaralda | Apr-10 | 7 |  | . |
| Risaralda | May-10 | 5 |  | . |
| … | … | … |  | … |
| Risaralda | May-15 | 6 |  | Medium |
| Risaralda | Jun-15 | 5 |  | Medium |
| Risaralda^a^ | Jul-15 | 10 |  | High |
| Risaralda | Aug-15 | 8 |  | High |
| Risaralda | Sep-15 | 11 | V | High |
| Risaralda | Oct-15 | 10 |  | High |
| Risaralda | Nov-15 | 8 |  | High |
| Risaralda | Dec-15 | 4 |  | . |
|  |  |  |  |  |
| **Department** | **Time** | **DIP** | **Outbreak peak** | **EWS** |
| Santander | Jun-07 | 23 |  | . |
| Santander^b^ | Jul-07 | 24 | V | Low |
| Santander | Aug-07 | 23 |  | Low |
| Santander | Sep-07 | 17 |  | . |
| Santander | Oct-07 | 13 |  | . |
| … | … | … |  | … |
| Santander | Nov-09 | 15 |  | Low |
| Santander | Dec-09 | 18 |  | Low |
| Santander^b^ | Jan-10 | 62 | V | High |
| Santander | Feb-10 | 16 |  | High |
| Santander | Mar-10 | 22 |  | High |
| Santander | Apr-10 | 19 |  | . |
| Santander | May-10 | 16 |  | . |
| … | … | … |  | … |
| Santander | Nov-12 | 14 |  | Low |
| Santander | Dec-12 | 14 |  | Low |
| Santander^a^ | Jan-13 | 23 |  | Medium |
| Santander | Feb-13 | 18 |  | Medium |
| Santander | Mar-13 | 20 |  | Medium |
| Santander | Apr-13 | 29 |  | Medium |
| Santander | May-13 | 37 | V | Medium |
| Santander | Jun-13 | 26 |  | Medium |
| Santander | Jul-13 | 35 |  | Medium |
| Santander | Aug-13 | 22 |  | Medium |
| Santander | Sep-13 | 24 |  | . |
| Santander | Oct-13 | 29 |  | . |
|  |  |  |  |  |
| **Department** | **Time** | **DIP** | **Outbreak peak** | **EWS** |
| Valle del Cauca | Nov-09 | 16 |  | Low |
| Valle del Cauca | Dec-09 | 24 |  | Low |
| Valle del Cauca^a^ | Jan-10 | 34 |  | High |
| Valle del Cauca | Feb-10 | 59 |  | High |
| Valle del Cauca | Mar-10 | 60 | V | High |
| Valle del Cauca | Apr-10 | 36 |  | High |
| Valle del Cauca | May-10 | 27 |  | High |
| Valle del Cauca | Jun-10 | 30 |  | . |
| Valle del Cauca | Jul-10 | 19 |  | . |
| … | … | … |  | … |
| Valle del Cauca | Nov-12 | 4 |  | . |
| Valle del Cauca | Dec-12 | 4 |  | . |
| Valle del Cauca^a^ | Jan-13 | 18 |  | Medium |
| Valle del Cauca | Feb-13 | 16 |  | Medium |
| Valle del Cauca | Mar-13 | 17 |  | Medium |
| Valle del Cauca | Apr-13 | 28 |  | Medium |
| Valle del Cauca | May-13 | 33 | V | Medium |
| Valle del Cauca | Jun-13 | 26 |  | Medium |
| Valle del Cauca | Jul-13 | 31 |  | Medium |
| Valle del Cauca | Aug-13 | 19 |  | . |
| Valle del Cauca | Sep-13 | 17 |  | . |

^a^ Correct warning signals 1~5 months ahead of time

^b^ Correct warning signals in the same month

^c^ Missed warning signals

^d^ Observed DIP

^e^ Based on three categorization of the elasticities: low level warning (0-50%), medium level warning (50-75%), and high level warning (75-100%).

^*^ This peak was manually added after checking the DIP trend

**Table S6(b)**

| **Department** | **SE** | **SP** | **PPV** | **NPV** |
| --- | --- | --- | --- | --- |
| Antioquia | 100% | 100% | 100% | 100% |
| Arauca | 100% | 100% | 100% | 100% |
| Boyaca | 100% | 100% | 100% | 100% |
| Cauca | 100% | 67% | 50% | 100% |
| Cundinamarca | 100% | 100% | 100% | 100% |
| Huila | 67% | 100% | 100% | 83% |
| Norte de Santander | 50% | 100% | 100% | 86% |
| Quindio | 100% | 100% | 100% | 100% |
| Risaralda | 50% | 100% | 100% | 86% |
| Santander | 100% | 100% | 100% | 100% |
| Valle del Cauca | 100% | 100% | 100% | 100% |

**Supplementary 7. EWS for 11 departments in 2015**

There were some inconsistent patterns observed between CRF and DIP due to the unexpected emergence of Zika which started being reported in 2015. The estimated FAR went up to 25% during 2015. As Zika is another viral disease caused by *Aedes aegypti,* the increased FAR might be explained by the growing number of reported Zika cases.

| **Department** | **Time** | **DIP** | **EWS** |
| --- | --- | --- | --- |
| Antioquia | Jan-15 | 6 | . |
| Antioquia | Feb-15 | 3 | . |
| Antioquia | Mar-15 | 2 | . |
| Antioquia | Apr-15 | 3 | . |
| Antioquia | May-15 | 2 | . |
| Antioquia | Jun-15 | 3 | . |
| Antioquia | Jul-15 | 6 | High |
| Antioquia | Aug-15 | 4 | High |
| Antioquia | Sep-15 | 9 | High |
| Antioquia | Oct-15 | 8 | High |
| Antioquia | Nov-15 | 6 | High |
| Antioquia | Dec-15 | 8 | High |
|  |  |  |  |
| **Department** | **Time** | **DIP** | **EWS** |
| Arauca | Jan-15 | 11 | . |
| Arauca | Feb-15 | 12 | . |
| Arauca | Mar-15 | 17 | Medium |
| Arauca | Apr-15 | 20 | Medium |
| Arauca | May-15 | 26 | Medium |
| Arauca | Jun-15 | 22 | Medium |
| Arauca | Jul-15 | 12 | High |
| Arauca | Aug-15 | 2 | . |
| Arauca | Sep-15 | 2 | . |
| Arauca | Oct-15 | 1 | . |
| Arauca | Nov-15 | 1 | . |
| Arauca | Dec-15 | 1 | . |
|  |  |  |  |
| **Department** | **Time** | **DIP** | **EWS** |
| Boyaca | Jan-15 | 2 | High |
| Boyaca | Feb-15 | 4 | High |
| Boyaca | Mar-15 | 3 | High |
| Boyaca | Apr-15 | 4 | High |
| Boyaca | May-15 | 8 | High |
| Boyaca | Jun-15 | 5 | . |
| Boyaca | Jul-15 | 6 | . |
| Boyaca | Aug-15 | 4 | . |
| Boyaca | Sep-15 | 5 | . |
| Boyaca | Oct-15 | 2 | . |
| Boyaca | Nov-15 | 1 | . |
| Boyaca | Dec-15 | 0 | . |
|  |  |  |  |
| **Department** | **Time** | **DIP** | **EWS** |
| Cauca | Jan-15 | 2 | . |
| Cauca | Feb-15 | 2 | High |
| Cauca | Mar-15 | 1 | High |
| Cauca | Apr-15 | 1 | High |
| Cauca | May-15 | 1 | . |
| Cauca | Jun-15 | 1 | . |
| Cauca | Jul-15 | 1 | . |
| Cauca | Aug-15 | 1 | . |
| Cauca | Sep-15 | 1 | High |
| Cauca | Oct-15 | 2 | High |
| Cauca | Nov-15 | 2 | High |
| Cauca | Dec-15 | 5 | High |
|  |  |  |  |
| **Department** | **Time** | **DIP** | **EWS** |
| Cundinamarca | Jan-15 | 3 | High |
| Cundinamarca | Feb-15 | 1 | High |
| Cundinamarca | Mar-15 | 1 | . |
| Cundinamarca | Apr-15 | 1 | . |
| Cundinamarca | May-15 | 1 | . |
| Cundinamarca | Jun-15 | 1 | . |
| Cundinamarca | Jul-15 | 1 | . |
| Cundinamarca | Aug-15 | 1 | . |
| Cundinamarca | Sep-15 | 1 | . |
| Cundinamarca | Oct-15 | 1 | . |
| Cundinamarca | Nov-15 | 1 | . |
| Cundinamarca | Dec-15 | 1 | High |
|  |  |  |  |
| **Department** | **Time** | **DIP** | **EWS** |
| Huila | Jan-15 | 20 | . |
| Huila | Feb-15 | 15 | . |
| Huila | Mar-15 | 12 | . |
| Huila | Apr-15 | 13 | . |
| Huila | May-15 | 11 | High |
| Huila | Jun-15 | 14 | . |
| Huila | Jul-15 | 19 | High |
| Huila | Aug-15 | 12 | High |
| Huila | Sep-15 | 19 | High |
| Huila | Oct-15 | 10 | . |
| Huila | Nov-15 | 13 | . |
| Huila | Dec-15 | 12 | . |
|  |  |  |  |
| **Department** | **Time** | **DIP** | **EWS** |
| Norte de Santander | Jan-15 | 9 | . |
| Norte de Santander | Feb-15 | 9 | Medium |
| Norte de Santander | Mar-15 | 7 | Medium |
| Norte de Santander | Apr-15 | 13 | Medium |
| Norte de Santander | May-15 | 14 | Medium |
| Norte de Santander | Jun-15 | 14 | Medium |
| Norte de Santander | Jul-15 | 18 | High |
| Norte de Santander | Aug-15 | 13 | High |
| Norte de Santander | Sep-15 | 17 | High |
| Norte de Santander | Oct-15 | 12 | . |
| Norte de Santander | Nov-15 | 12 | . |
| Norte de Santander | Dec-15 | 8 | . |
|  |  |  |  |
| **Department** | **Time** | **DIP** | **EWS** |
| Quindio | Jan-15 | 28 | Medium |
| Quindio | Feb-15 | 30 | Medium |
| Quindio | Mar-15 | 33 | Medium |
| Quindio | Apr-15 | 59 | Medium |
| Quindio | May-15 | 42 | Medium |
| Quindio | Jun-15 | 38 | Medium |
| Quindio | Jul-15 | 46 | High |
| Quindio | Aug-15 | 22 | . |
| Quindio | Sep-15 | 26 | . |
| Quindio | Oct-15 | 14 | . |
| Quindio | Nov-15 | 11 | . |
| Quindio | Dec-15 | 4 | . |
|  |  |  |  |
| **Department** | **Time** | **DIP** | **EWS** |
| Risaralda | Jan-15 | 3 | . |
| Risaralda | Feb-15 | 3 | . |
| Risaralda | Mar-15 | 5 | Medium |
| Risaralda | Apr-15 | 6 | Medium |
| Risaralda | May-15 | 6 | Medium |
| Risaralda | Jun-15 | 5 | Medium |
| Risaralda | Jul-15 | 10 | High |
| Risaralda | Aug-15 | 8 | High |
| Risaralda | Sep-15 | 11 | High |
| Risaralda | Oct-15 | 10 | High |
| Risaralda | Nov-15 | 8 | High |
| Risaralda | Dec-15 | 4 | . |
|  |  |  |  |
| **Department** | **Time** | **DIP** | **EWS** |
| Santander | Jan-15 | 12 | . |
| Santander | Feb-15 | 9 | . |
| Santander | Mar-15 | 10 | High |
| Santander | Apr-15 | 15 | High |
| Santander | May-15 | 12 | High |
| Santander | Jun-15 | 9 | High |
| Santander | Jul-15 | 12 | High |
| Santander | Aug-15 | 8 | . |
| Santander | Sep-15 | 9 | . |
| Santander | Oct-15 | 7 | . |
| Santander | Nov-15 | 7 | . |
| Santander | Dec-15 | 5 | . |
|  |  |  |  |
| **Department** | **Time** | **DIP** | **EWS** |
| Valle del Cauca | Jan-15 | 14 | Medium |
| Valle del Cauca | Feb-15 | 14 | Medium |
| Valle del Cauca | Mar-15 | 12 | Medium |
| Valle del Cauca | Apr-15 | 13 | Medium |
| Valle del Cauca | May-15 | 10 | Medium |
| Valle del Cauca | Jun-15 | 8 | . |
| Valle del Cauca | Jul-15 | 10 | . |
| Valle del Cauca | Aug-15 | 7 | . |
| Valle del Cauca | Sep-15 | 8 | . |
| Valle del Cauca | Oct-15 | 6 | . |
| Valle del Cauca | Nov-15 | 6 | . |
| Valle del Cauca | Dec-15 | 11 | High |

**Supplementary 8. Adjustment for case-underreporting**

Our study attempted to adjust the crude cases reported by SIVIGILA by the proportions of municipalities who were not responsive for each department. The assumption used in this study, “a non-responsive municipality would have the average number of cases per responsive municipality of that department”, may not be perfect, but this is a sensible assumption based on the following reason:

Considering the nature of the EWS model using count models and the elasticity of the function, what is important is a consistent pattern of case-reporting by department over time. In other words, a consistent underreporting or consistent overreporting would not affect the performance of the EWS model because this would merely shift the entire DIP curve either downwards (underreporting) or upwards (overreporting). Thus, what we wanted to be cautious was any lack of consistency over time in terms of case-reporting.

Supplementary Figure 8 depicts the trend of the crude DIP and the adjusted DIP over time. As described in the main text, Colombia experienced a nationwide dengue outbreak in 2010. For example, in Santander, while there was no big difference between the crude and adjusted DIPs during non-epidemic periods, a substantial difference between the two was observed in 2010. This clearly indicates a lack of consistency in terms of case-reporting during the outbreak period. Likewise, the crude and adjusted DIPs appeared to be similar during non-epidemic seasons in Norte de Santander, but again discrepancies were observed during the two outbreak periods (2010 and 2013).

Given that case-reporting patterns among all municipalities were reasonably consistent (no significant discrepancy between crude and adjusted DIPs) during non-epidemic periods and discrepancies only occur during outbreak periods, it is reasonable to assume that there were more difficulties for some municipalities on resource allocation at the local health facility level during the epidemic periods, impeding a consistent pattern in the case-reporting system from municipality-level heath facilities, even if non-responsive municipalities were experiencing the similar emergency situations with other responsive municipalities.

**Supplementary Figure 8. Antioquia**
