# Supplementary figures and images for: Early warning signal for dengue outbreaks and identification of high risk areas for dengue fever in Colombia using climate and non-climate datasets
Source: BMC Infect Dis. 2017 Jul 10;17:480. doi: 10.1186/s12879-017-2577-4 (PMC5504639; doi:10.1186/s12879-017-2577-4)

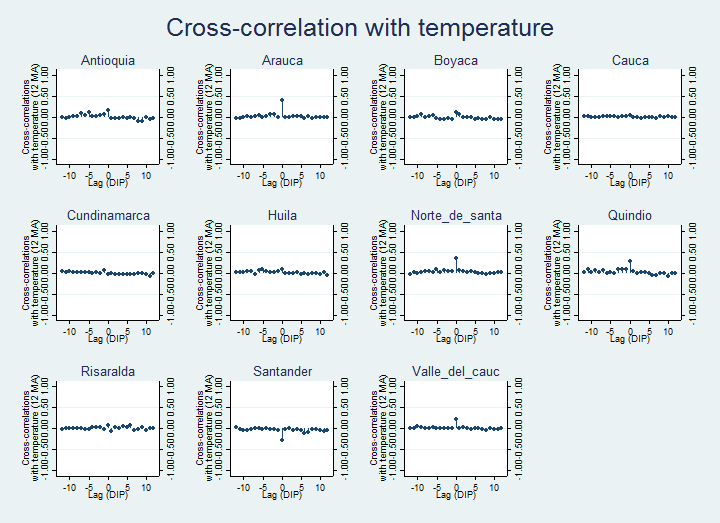

Supplement: Supplementary file 1 — Supplementary 1.Cross-correlograms of climate datasets and DIP. Supplementary 2. Model specifications. Supplementary 3. Climate factors and DIP over time by department. Supplementary 4. The CRF index and DIP over time by department. Supplementary 5. Identification of high risk areas for dengue fever. Supplementary 6. EWS for 11 departments during the study period. Supplementary 7. EWS for 11 departments in 2015. (ZIP 2317 kb) [file 12879_2017_2577_MOESM1_ESM.zip › Supplementary1(a)R2.tif]

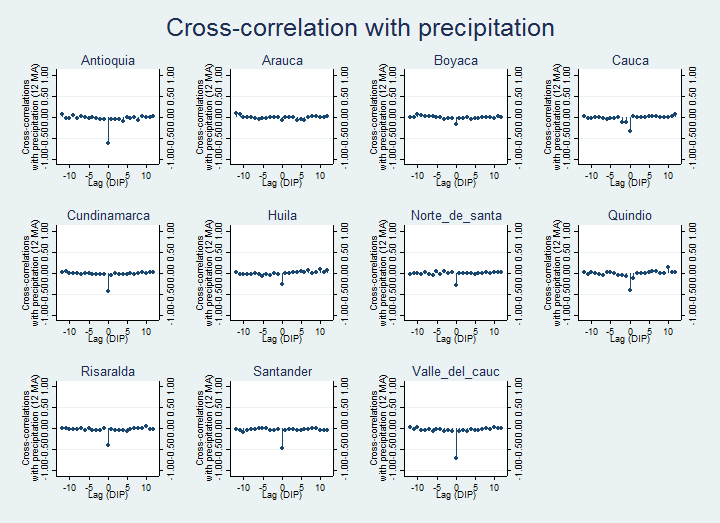

Supplement: Supplementary file 1 — Supplementary 1.Cross-correlograms of climate datasets and DIP. Supplementary 2. Model specifications. Supplementary 3. Climate factors and DIP over time by department. Supplementary 4. The CRF index and DIP over time by department. Supplementary 5. Identification of high risk areas for dengue fever. Supplementary 6. EWS for 11 departments during the study period. Supplementary 7. EWS for 11 departments in 2015. (ZIP 2317 kb) [file 12879_2017_2577_MOESM1_ESM.zip › Supplementary1(b)R2.tif]

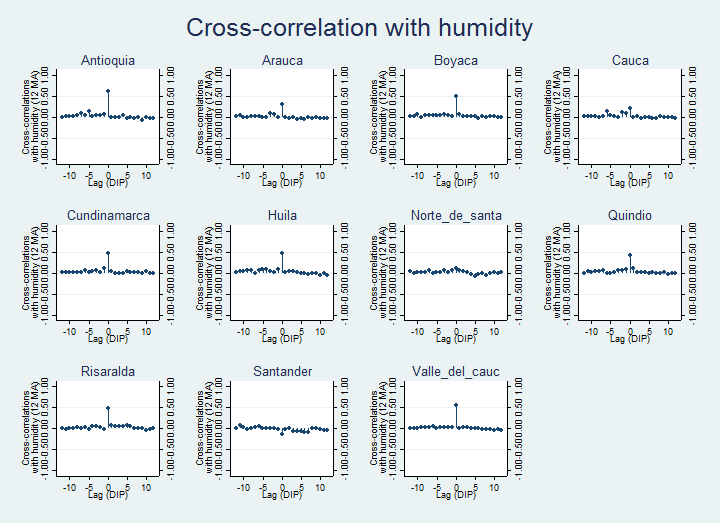

Supplement: Supplementary file 1 — Supplementary 1.Cross-correlograms of climate datasets and DIP. Supplementary 2. Model specifications. Supplementary 3. Climate factors and DIP over time by department. Supplementary 4. The CRF index and DIP over time by department. Supplementary 5. Identification of high risk areas for dengue fever. Supplementary 6. EWS for 11 departments during the study period. Supplementary 7. EWS for 11 departments in 2015. (ZIP 2317 kb) [file 12879_2017_2577_MOESM1_ESM.zip › Supplementary1(c)R2.tif]

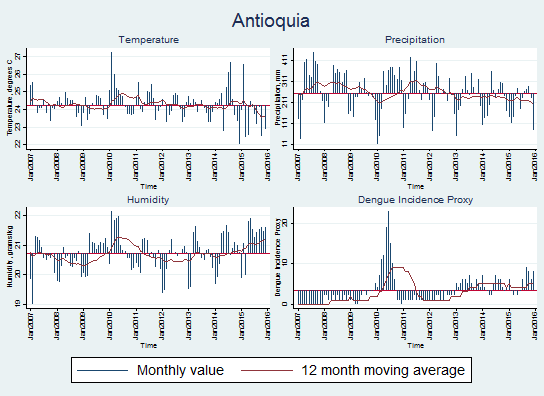

Supplement: Supplementary file 1 — Supplementary 1.Cross-correlograms of climate datasets and DIP. Supplementary 2. Model specifications. Supplementary 3. Climate factors and DIP over time by department. Supplementary 4. The CRF index and DIP over time by department. Supplementary 5. Identification of high risk areas for dengue fever. Supplementary 6. EWS for 11 departments during the study period. Supplementary 7. EWS for 11 departments in 2015. (ZIP 2317 kb) [file 12879_2017_2577_MOESM1_ESM.zip › Supplementary3(a)_AntioquiaR2.tif]

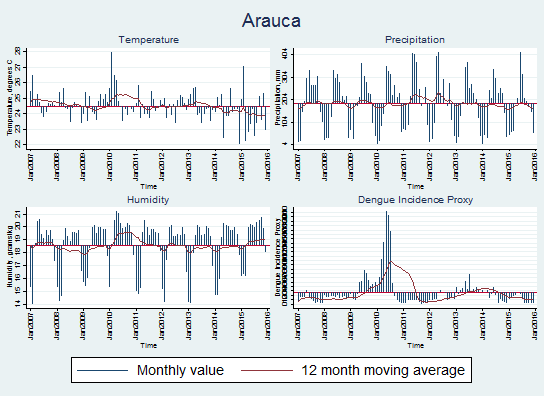

Supplement: Supplementary file 1 — Supplementary 1.Cross-correlograms of climate datasets and DIP. Supplementary 2. Model specifications. Supplementary 3. Climate factors and DIP over time by department. Supplementary 4. The CRF index and DIP over time by department. Supplementary 5. Identification of high risk areas for dengue fever. Supplementary 6. EWS for 11 departments during the study period. Supplementary 7. EWS for 11 departments in 2015. (ZIP 2317 kb) [file 12879_2017_2577_MOESM1_ESM.zip › Supplementary3(b)_AraucaR2.tif]

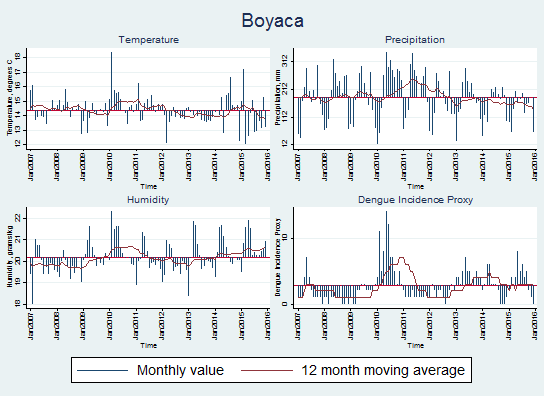

Supplement: Supplementary file 1 — Supplementary 1.Cross-correlograms of climate datasets and DIP. Supplementary 2. Model specifications. Supplementary 3. Climate factors and DIP over time by department. Supplementary 4. The CRF index and DIP over time by department. Supplementary 5. Identification of high risk areas for dengue fever. Supplementary 6. EWS for 11 departments during the study period. Supplementary 7. EWS for 11 departments in 2015. (ZIP 2317 kb) [file 12879_2017_2577_MOESM1_ESM.zip › Supplementary3(c)_BoyacaR2.tif]

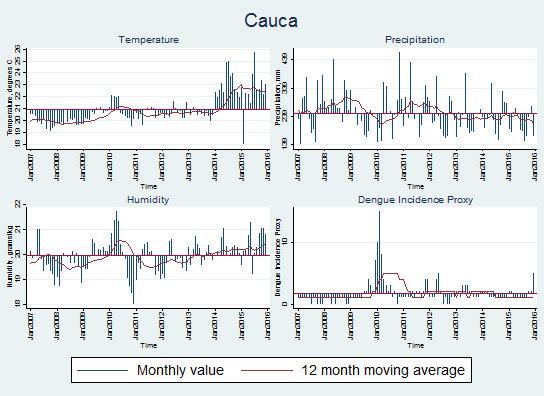

Supplement: Supplementary file 1 — Supplementary 1.Cross-correlograms of climate datasets and DIP. Supplementary 2. Model specifications. Supplementary 3. Climate factors and DIP over time by department. Supplementary 4. The CRF index and DIP over time by department. Supplementary 5. Identification of high risk areas for dengue fever. Supplementary 6. EWS for 11 departments during the study period. Supplementary 7. EWS for 11 departments in 2015. (ZIP 2317 kb) [file 12879_2017_2577_MOESM1_ESM.zip › Supplementary3(d)_CaucaR2.tif]

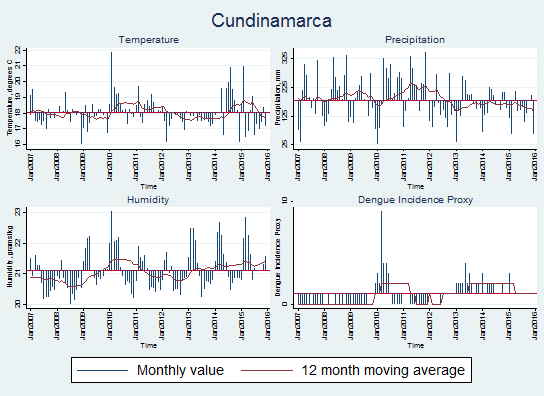

Supplement: Supplementary file 1 — Supplementary 1.Cross-correlograms of climate datasets and DIP. Supplementary 2. Model specifications. Supplementary 3. Climate factors and DIP over time by department. Supplementary 4. The CRF index and DIP over time by department. Supplementary 5. Identification of high risk areas for dengue fever. Supplementary 6. EWS for 11 departments during the study period. Supplementary 7. EWS for 11 departments in 2015. (ZIP 2317 kb) [file 12879_2017_2577_MOESM1_ESM.zip › Supplementary3(e)_CundinamarcaR2.tif]

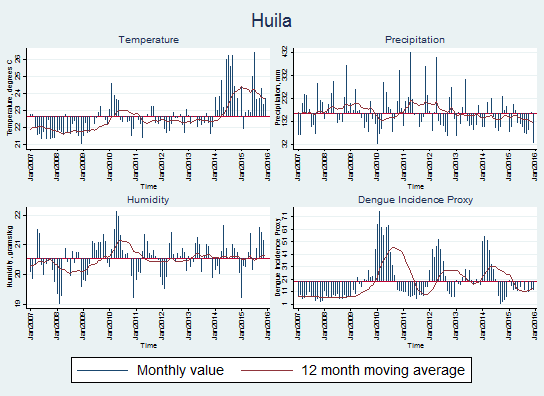

Supplement: Supplementary file 1 — Supplementary 1.Cross-correlograms of climate datasets and DIP. Supplementary 2. Model specifications. Supplementary 3. Climate factors and DIP over time by department. Supplementary 4. The CRF index and DIP over time by department. Supplementary 5. Identification of high risk areas for dengue fever. Supplementary 6. EWS for 11 departments during the study period. Supplementary 7. EWS for 11 departments in 2015. (ZIP 2317 kb) [file 12879_2017_2577_MOESM1_ESM.zip › Supplementary3(f)_HuilaR2.tif]

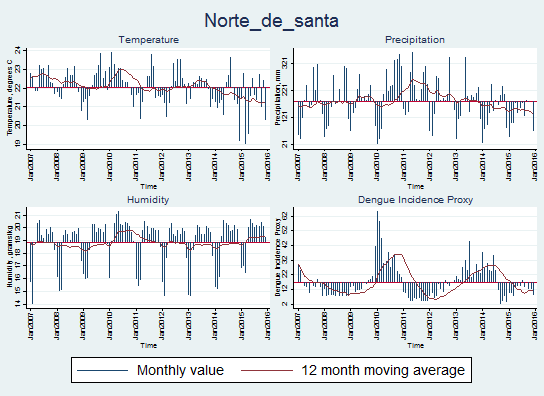

Supplement: Supplementary file 1 — Supplementary 1.Cross-correlograms of climate datasets and DIP. Supplementary 2. Model specifications. Supplementary 3. Climate factors and DIP over time by department. Supplementary 4. The CRF index and DIP over time by department. Supplementary 5. Identification of high risk areas for dengue fever. Supplementary 6. EWS for 11 departments during the study period. Supplementary 7. EWS for 11 departments in 2015. (ZIP 2317 kb) [file 12879_2017_2577_MOESM1_ESM.zip › Supplementary3(g)_Norte_de_santaR2.tif]

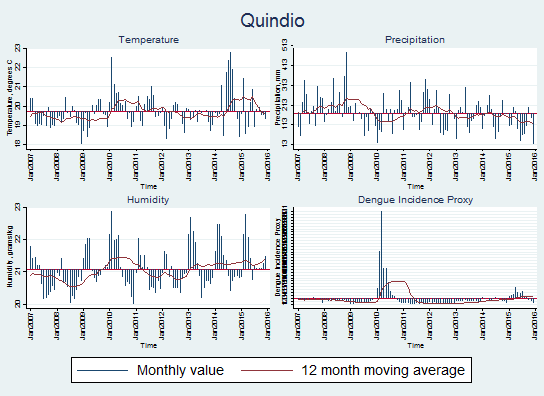

Supplement: Supplementary file 1 — Supplementary 1.Cross-correlograms of climate datasets and DIP. Supplementary 2. Model specifications. Supplementary 3. Climate factors and DIP over time by department. Supplementary 4. The CRF index and DIP over time by department. Supplementary 5. Identification of high risk areas for dengue fever. Supplementary 6. EWS for 11 departments during the study period. Supplementary 7. EWS for 11 departments in 2015. (ZIP 2317 kb) [file 12879_2017_2577_MOESM1_ESM.zip › Supplementary3(h)_QuindioR2.tif]

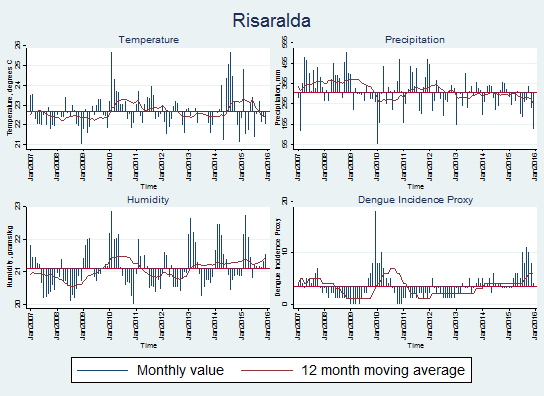

Supplement: Supplementary file 1 — Supplementary 1.Cross-correlograms of climate datasets and DIP. Supplementary 2. Model specifications. Supplementary 3. Climate factors and DIP over time by department. Supplementary 4. The CRF index and DIP over time by department. Supplementary 5. Identification of high risk areas for dengue fever. Supplementary 6. EWS for 11 departments during the study period. Supplementary 7. EWS for 11 departments in 2015. (ZIP 2317 kb) [file 12879_2017_2577_MOESM1_ESM.zip › Supplementary3(i)_RisaraldaR2.tif]

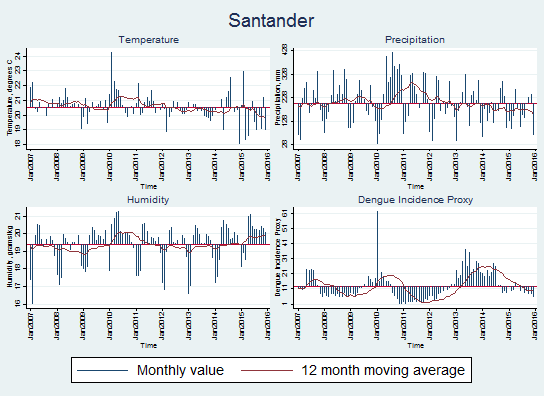

Supplement: Supplementary file 1 — Supplementary 1.Cross-correlograms of climate datasets and DIP. Supplementary 2. Model specifications. Supplementary 3. Climate factors and DIP over time by department. Supplementary 4. The CRF index and DIP over time by department. Supplementary 5. Identification of high risk areas for dengue fever. Supplementary 6. EWS for 11 departments during the study period. Supplementary 7. EWS for 11 departments in 2015. (ZIP 2317 kb) [file 12879_2017_2577_MOESM1_ESM.zip › Supplementary3(j)_SantanderR2.tif]

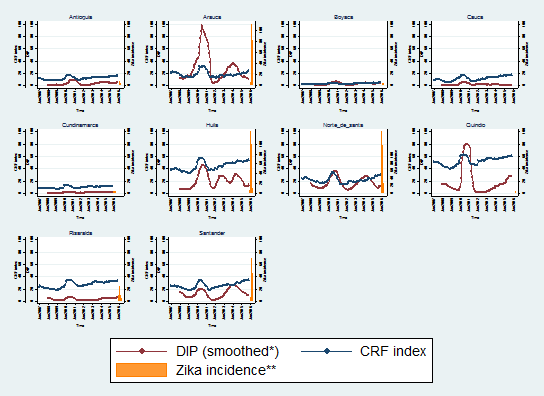

Supplement: Supplementary file 1 — Supplementary 1.Cross-correlograms of climate datasets and DIP. Supplementary 2. Model specifications. Supplementary 3. Climate factors and DIP over time by department. Supplementary 4. The CRF index and DIP over time by department. Supplementary 5. Identification of high risk areas for dengue fever. Supplementary 6. EWS for 11 departments during the study period. Supplementary 7. EWS for 11 departments in 2015. (ZIP 2317 kb) [file 12879_2017_2577_MOESM1_ESM.zip › Supplementary4R2.tif]

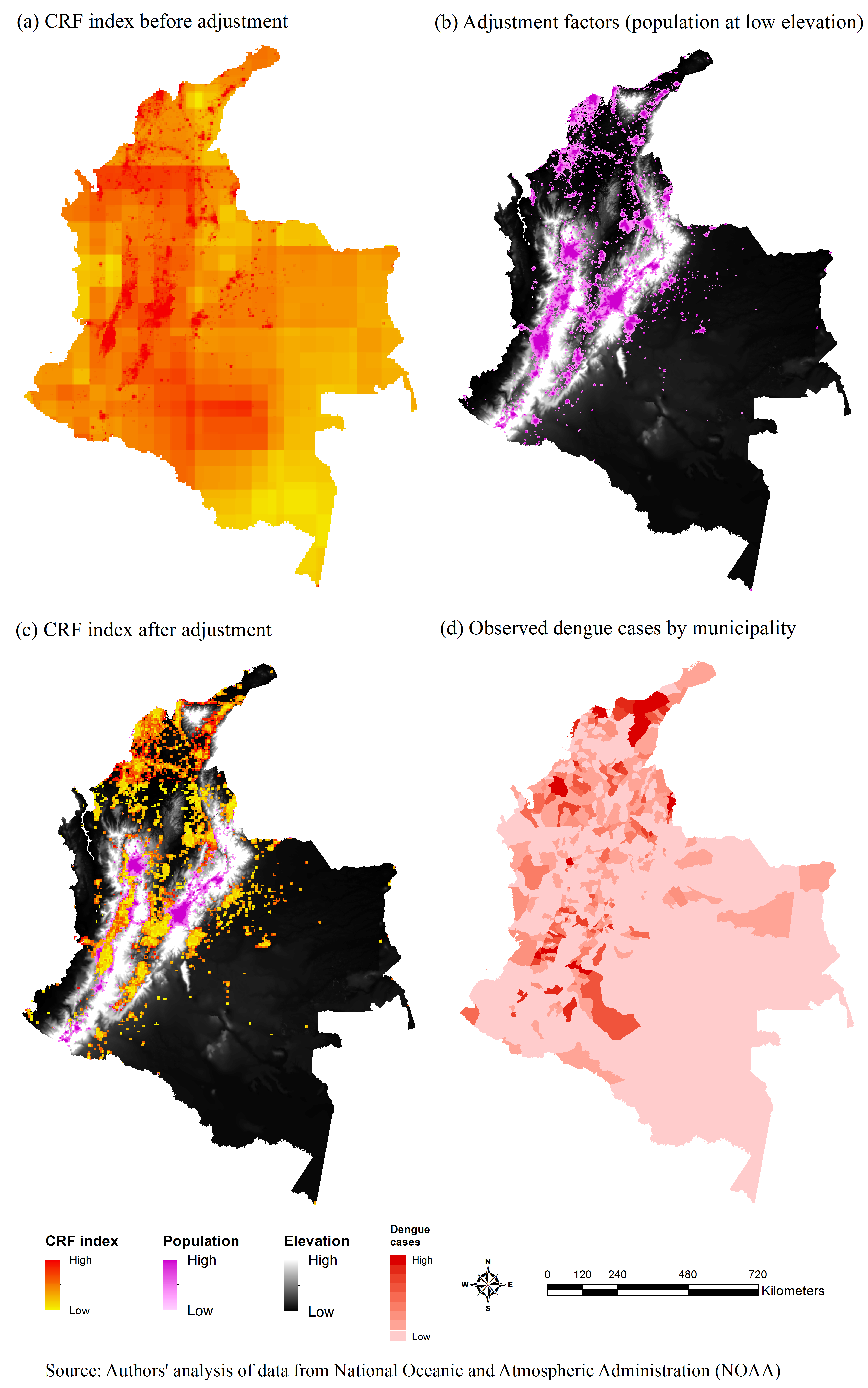

Supplement: Supplementary file 1 — Supplementary 1.Cross-correlograms of climate datasets and DIP. Supplementary 2. Model specifications. Supplementary 3. Climate factors and DIP over time by department. Supplementary 4. The CRF index and DIP over time by department. Supplementary 5. Identification of high risk areas for dengue fever. Supplementary 6. EWS for 11 departments during the study period. Supplementary 7. EWS for 11 departments in 2015. (ZIP 2317 kb) [file 12879_2017_2577_MOESM1_ESM.zip › Supplementary5R2.tif]

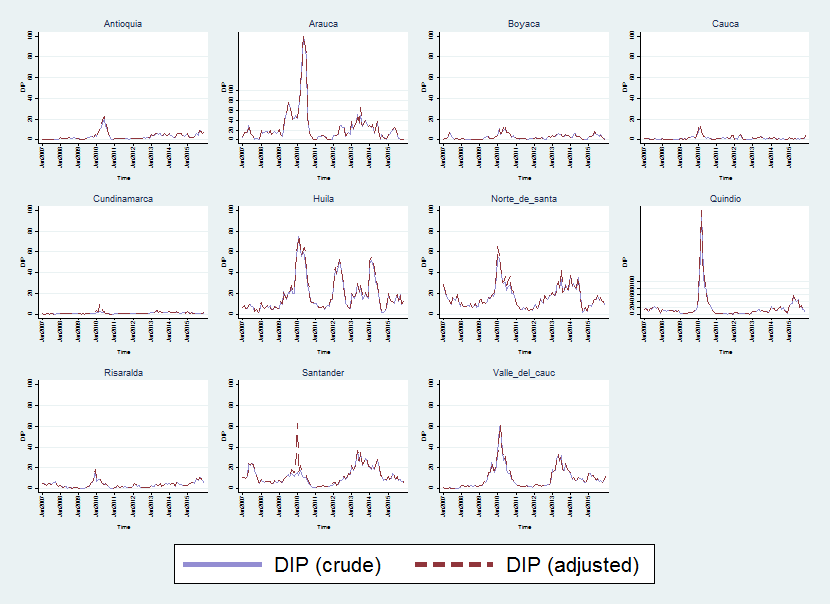

Supplement: Supplementary file 1 — Supplementary 1.Cross-correlograms of climate datasets and DIP. Supplementary 2. Model specifications. Supplementary 3. Climate factors and DIP over time by department. Supplementary 4. The CRF index and DIP over time by department. Supplementary 5. Identification of high risk areas for dengue fever. Supplementary 6. EWS for 11 departments during the study period. Supplementary 7. EWS for 11 departments in 2015. (ZIP 2317 kb) [file 12879_2017_2577_MOESM1_ESM.zip › Supplementary8R2.tif]
